# Prediction of high-grade cervical intraepithelial neoplasia in cytologically normal women by human papillomavirusesting

**DOI:** 10.1054/bjoc.2000.1491

**Published:** 2000-11-22

**Authors:** F Carozzi, G Ronco, M Confortini, D Noferini, C Maddau, S Ciatto, N Segnan

**Affiliations:** Cytology Unit, Centre for Cancer Study and Prevention (CSPO), Firenze, Italy; Cancer Epidemiology Unit, ASL TO1, Centre for Cancer Epidemiology and Prevention (CPO), Torino, Italy; Department of Diagnostic Medical Imaging, Centre for Cancer Study and Prevention (CSPO), Florence, Italy

## Abstract

Human papillomavirus (HPV) testing has been suggested for primary screening of cervical cancer. Prediction of future high-grade cervical lesions is crucial for effectiveness and cost. We performed a case control study in a retrospective cohort of women with at least two cervical smears, all but the last one being negative, from the organized cervical screening programme in Florence, Italy. We searched for high-risk HPV in all previous, archival, smears from cases (new histologically confirmed cervical intraepithelial neoplasia (CIN) grade II or worse) and in one previous smear from each control (last smear cytologically normal, matched by age and interval (latency) from last smear). We applied polymerase chain reaction (PCR), and the b-globin gene was used as a DNA preservation marker. High-risk HPV was identified in 71/92 (77.17%) previous smears from 79 cases and 17/332 controls (5.12%). The odds ratio (OR) was 63.76 (95% CI 30.57–132.96). Among cases the proportion of HPV-positive smears declined slightly with increasing latency. Among cases, HPV was found in 81.24% (95% CI 69.93–88.96%) of smears with latency < 4 years and in 67.80% (95% CI 47.72–82.93%) of those taken at longer intervals, up to 6 years. These findings suggest that testing for high-risk HPV allows predicting 80% of CINII/III 3 years before the cytological diagnosis and two thirds 6 years before. They also suggest that testing women negative for high-risk HPV at longer interval and strictly following-up women who are HPV positive could be an effective strategy for cervical cancer screening. © 2000 Cancer Research Campaign http://www.bjcancer.com


*British Journal of Cancer* (2000) **83**(11), 1462–1467
© 2000 Cancer Research Campaign
doi: 10.1054/ bjoc.2000.1491, available online at http://www.idealibrary.com on IDEAL®

### Prediction of high-grade cervical intraepithelial neoplasia in cytologically normal women by human papillomavirus testing

**F Carozzi[1], G Ronco[2], M Confortini[1], D Noferini[1], C Maddau[1], S Ciatto[3] and N Segnan[2]**

[1]Cytology Unit, Centre for Cancer Study and Prevention (CSPO), Firenze, Italy; [2]Cancer Epidemiology Unit, ASL TO1, Centre for Cancer Epidemiology and Prevention (CPO), Torino, Italy; [3]Department of Diagnostic Medical Imaging, Centre for Cancer Study and Prevention (CSPO), Florence, Italy

Summary Human papillomavirus (HPV) testing has been suggested for primary screening of cervical cancer. Prediction of future high-grade cervical lesions is crucial for effectiveness and cost. We performed a case control study in a retrospective cohort of women with at least two cervical smears, all but the last one being negative, from the organized cervical screening programme in Florence, Italy. We searched for high-risk HPV in all previous, archival, smears from cases (new histologically confirmed cervical intraepithelial neoplasia (CIN) grade II or worse) and in one previous smear from each control (last smear cytologically normal, matched by age and interval (latency) from last smear). We applied polymerase chain reaction (PCR), and the b-globin gene was used as a DNA preservation marker. High-risk HPV was identified in 71/92 (77.17%) previous smears from 79 cases and 17/332 controls (5.12%). The odds ratio (OR) was 63.76 (95% CI 30.57–132.96). Among cases the proportion of HPV-positive smears declined slightly with increasing latency. Among cases, HPV was found in 81.24% (95% CI 69.93–88.96%) of smears with latency < 4 years and in 67.80% (95% CI 47.72–82.93%) of those taken at longer intervals, up to 6 years. These findings suggest that testing for high-risk HPV allows predicting 80% of CINII/III 3 years before the cytological diagnosis and two thirds 6 years before. They also suggest that testing women negative for high-risk HPV at longer interval and strictly following-up women who are HPV positive could be an effective strategy for cervical cancer screening. © 2000 Cancer Research Campaign http://www.bjcancer.com

Infection by some types of human papillomavirus (HPV) has been established to be causally related both to cervical cancer and to cervical intraepithelial neoplasia (CIN) (IARC, 1995). Existing evidence derives mainly from cross-sectional case-control studies that found high frequencies of HPV infection in invasive cancers and in CIN at the time of diagnosis and low frequencies in controls with negative cytology (see references in IARC, 1995). On this basis HPV DNA testing has been suggested as a possible primary test, with or without conventional cytology, for the screening of cervical cancer (Meijer et al, 1992; Solomon, 1993; Richart, 1995; Cuzick, 2000).

The effectiveness and cost of HPV testing as a primary screening method depends on its ability to predict a future diagnosis of CIN. In particular, if HPV-negative women were at very low risk of developing high-grade CIN for many years, this would allow screening them at longer intervals and concentrating on HPV-positive subjects.

Some follow-up studies provide evidence that persistence of high-risk HPV infection is needed for progression to high-grade lesions of women with abnormal cytology (Ho et al, 1995; Remmink et al, 1995; Nobbenhuis et al, 1999). One population study on the risk of squamous intraepithelial lesions (SIL) (Liaw et al, 1999) and one of invasive cancer (Wallin et al, 1999) subsequent to HPV detection in cytologically normal women have recently been published. Other, previous, studies were based on small number of cases (Walboomers et al, 1995; Rozendaal et al, 1996). Similar follow-up studies performed before 1995 are reviewed in the IARC monograph on HPV (IARC, 1995). The number of observed cases was always small (never reached 30) and follow-up duration generally limited. None of them used PCR for HPV detection: methods of more limited accuracy like dot-blot or filter in situ hybridization were more commonly used. In some cases selected populations (e.g. recruited in sexually transmitted disease clinics) were studied.

We performed a case-control study by testing previous cytologically negative smears for HPV DNA, in order to estimate the value of HPV testing in predicting histologically confirmed high-grade CIN at different time intervals.

### MATERIALS AND METHODS

The setting was the organized screening programme for cervical cancer in Florence (Italy) that has been active for many years (Palli et al, 1990). Cervical cytology is the primary screening method and is recommended every third year. Women aged 25 to 64 years who are found not to have had cervical cytology within 3 years are invited. Women outside this age class can have spontaneous cytology: some 7% of screened women are younger than 25 years. The screening interval can be shorter than 3 years when a woman performs tests upon request: the average interval between screening exams is 2.8 years. Cytology is classified according to the Bethesda system, using standard forms. Women are referred for colposcopy if cytology shows low-grade squamous intraepithelial lesion (LSIL) or more severe abnormalities. Cases of atypical cells of undetermined significance (ASCUS) were routinely referred for colposcopy until October 1996, while after that date

*Received 7 March 2000*
*Revised 22 June 2000*
*Accepted 10 August 2000*

*Correspondence to:* G Ronco they were advised to repeat cytology in 6 months. Smear interpretation and colposcopy are centralized. All cytological and histological results of women screened within the organized programme have been registered on a computerized database since 1980. Some 80% of smears performed by Florence inhabitants are interpreted in the centralized laboratory. Quality assurance programmes, including circulation of standard sets of smears (Confortini et al, 1993) and peer review of abnormal smears (Palli et al, 1993) are routinely implemented. Biopsies performed outside the organized programme are routinely obtained through the local cancer registry.

All women screened, independently of invitation, were considered. Of these, the eligible women were those who (a) had 2 or more smears, (b) had the last cytology (index smear) performed between January 1992 and March 1998, (c) were never referred for colposcopy at previous cytology tests and had no history of CIN.

Cases were eligible subjects with a histologically confirmed diagnosis of CIN II or more severe lesion after the index smear. All smears taken within 6 years before the index smear (and in any case after 1989), but not the index smear itself, were retrieved and independently reviewed by 3 of the authors (MC, FC, CM). Smears classified as SIL or more severe at review were excluded. Potential controls were women not referred for colposcopy at the last (index) smear. One previous smear was retrieved for each control. We sampled the eligible population for 3 control smears per case smear, matched to cases by age (±3 years), by year of the index smear, and by interval from the index smear (±1 year).

DNA from a pool of major interest high-risk HPV types (16, 18, 31, 33, 52, 58) was searched for on retrieved smears.

### Molecular analysis

In order to remove cells from archived slides, Pap smears were soaked in xylene for 2 to 6 days until the coverslips were removed, then destained in a solution of ethanol 70% and HCl 0.1% for 5–10 minutes. Cells were scraped from the slide with a clean new slide and transferred into an Eppendorf tube. To maximize the yield, DNA isolation was performed using a method based on a high capacity silica matrix (Roda Husman de et al, 1995) in the presence of the chaotropic agent guanidium thiociate (Quiagen tissue kit).

To analyse the quality of the target DNA, the samples were screened by PCR using β-globin specific primers (PC04-GH20) spanning 268 base pairs (bp). Only samples that tested positive for β-globin were further analysed.

Testing for human papillomavirus was done by PCR, using consensus pU-1M and pU-2R primers (Fujinaga et al, 1991) within the E6-E7 region of high-risk HPV types 16, 18, 31, 33, 52 and 58. This test provides a simple accurate mean of detecting a broad range of high-risk HPVs and has been clinically validated (Fujinaga et al, 1991). The pU-1M/pU-2R primers pair yielded a PCR product of 233 to 268 bp when DNA of HPV types 16, 18, 31, 33, 52 or 58 is present in the sample.

The sample (5 μl) was added to 40 μl of PCR mixture (BIOLINE Diagnostici s.r.l., Turin, Italy) containing dNTP, $MgCl_2$ and primers in PCR buffer. During the first denaturation step, 5 μl of Taq Polymerase (2.5 units) were added (hot start technique). The PCR reaction was performed in a total volume of 50 μl. The termal cycling conditions were 94˚C for 5 min, then 40 cycles at 94˚C for 1 min, 55˚C for 1 min, 72˚C for 1 min and finally 72˚C for 5 min, using Omnigene Thermal Cycler (Hybaid Ltd).

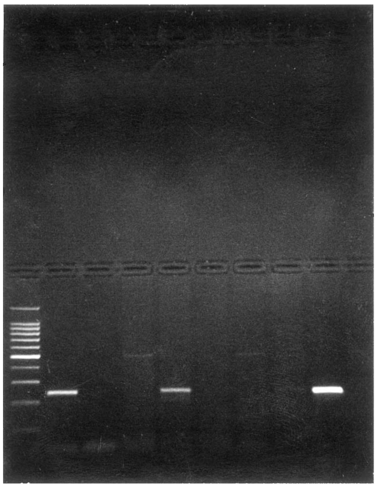

**Figure 1** E6–E7 amplification products. DNA was electrophoresed on 2% agarose gel, stained by ethidium bromide, visualized and photographed under UV light. Lane MW: molecular weight, 100 bp DNA ladder (Genenco, Florence, Italy). Lane C+: positive control, DNA from CaSki cells. Lane C–: negative control, sterile water. Lanes 1 to 7: DNA from clinical specimens. Samples 2 and 6 were positive for high-risk HPV DNA; samples 1, 3, 4, 5, 7 were negative.

In each run of amplification analysis a negative control and a positive control were included, to verify the results and to avoid false positive signals due to contamination. PCR positive control consisted of extracted DNA from the CaSki cell line which contains HPV 16 (Kreatech Diagnostics). Sterile water was used as the negative control. Regarding sample, the analyst was not aware of case/control status.

The amplified products were applied to 2% agarose gel containing ethidium bromide and electrophoresed. The DNA were visualized under ultraviolet (UV) transillumination. A band of 233–268 bp size indicated a positive samples for a HPV from the high risk pool (16, 18, 31, 33, 52, 58) (Figure 1).

### Statistical methods

Only smears in which β-globin DNA was identifiable were used for the analysis. Smears were the statistical units in every analysis. If a case had smears taken at different intervals before the index smear, each of them provided information on the subject's HPV status at the corresponding interval. As observations from the same subject are possibly correlated, test results cannot be assumed to be independent. Therefore test statistics and confidence intervals are not correct with standard procedures. We took this into account by use of Generalized Estimating Equations (GEE) (Zeger and Liang, 1986).

As multiple case and control smears had the same matching covariable values, we computed the OR between case/control status and the result of HPV testing, adjusting (Mantel-Heanszel OR) with strata that grouped case and control smears with the same combination of covariables (including exact age) (Brookmeyer et al, 1986). The same OR was also computed by unconditional logistic regression, adjusting for age class and for interval from the index smear and applying GEE in order to have correct confidence intervals.

We studied a possible modification of HPV effect by test-index smear interval by adding a HPV × interval interaction

© 2000 Cancer Research Campaign term. Interval was re-grouped in two classes (> 4 years and ≥ 4 years).

We studied the variations in the proportion of HPV-positive smears, by age and by interval between tested and index smears, within cases and controls, using unconditional logistic regression. For cases we applied GEE. GEE were also used in order to obtain correct confidence intervals for the proportion of HPV positive smears at intervals < 4 years and ≥ 4 years (Ronco and Biggeri, 1999).

### RESULTS

We retrieved 104 smears from cases and 350 from controls. Among case smears 5 were excluded because they were classified as SIL at review and 7 because β-globin DNA could not be identified. 18 control smears were also excluded for the latter reason. We identified β-globin DNA in 92 smears from 79 cases (15 CIN II, 59 CIN III, 5 invasive cancers) and in 332 smears from controls. A single study smear was available for 69 cases, 2 for 8 cases, 3 for one case and 4 for another. About half the case and control smears were taken at age ≥ 35 years (Table 1). Only 2 case and 7 control smears were taken before age 25. The mean interval between test and index smear was 2.5 years (Table 2).

Among case smears 71 (77.17%) were positive for high-risk HPV DNA, vs. 17 (5.12%) control smears, with an OR (adjusted for age class and interval between the test smear and the index smear) of 63.76 (95% CI 30.57–132.96). The corresponding Mantel-Haenszel OR, with strata grouping case and control smears with the same combination of matching covariables (including exact age) was 61.72. The OR was significantly higher (z robust = –2.26, $P$ = 0.024) for smears taken less than 4 years before the index one (OR = 102.94, 95% CI 42.63–250.72) than for those taken at longer interval (OR = 21.00, 95% CI 7.22–61.21).

Among cases, the proportion of HPV-positive smears showed little variation by age. Among controls there was a statistically not significant reduction from 5.6% in smears taken until 44 years of age to 2.3% in smears taken at older ages (Table 1).

Among cases, most smears remained HPV positive even 5–6 years before the index smear (Table 2). A slight decrease with an increasing test-index smear interval was not statistically significant (z robust for linear trend = –0.75, P = 0.453). High-risk HPV DNA was found in 81.24% (95% CI 69.93–88.96%) and 67.80% (95% CI 47.72–82.93%) of case smears, for intervals from the index smear of < 4 years and ≥ 4 years respectively.

In order to study the duration of HPV infection before a high-grade lesion arises, we also considered the midpoint between the index smear and the last previous negative cytology and we studied HPV presence as a function of time before it (Table 3). The picture changed only very slightly.

Previous smears from cases were reviewed before HPV testing. Some 30 smears were re-classified as ASCUS. When excluding them, the proportion of smears containing high-risk HPV DNA was 79.11% (95% CI 64.35–88.82%) and 57.96% (95% CI 36.83–76.53%) for intervals from index smear < 4 and ≥ 4 years, respectively. The duration of HPV infection before the start of CINII+ was also re-computed excluding smears classified as ASCUS at review if no further smear ‘within normal limits’ was present. Results (Table 3) were only slightly different from those based on all cases.

Among controls the proportion of HPV-positive smears was substantially constant across test-index smear intervals (Table 2) ($\chi^2$ for linear trend = –1.07, P = 0.301).

**Table 1** Distribution of case and control smears by age at smear-taking and presence of high-risk HPV

| Age at smear taking (years) | Case smears | | Control smears | |
|---|---|---|---|---|
| | Number of smears | No. (%) positive for high-risk HPV | Number of smears | No. (%) positive for high-risk HPV |
| < 35 | 49 | 37 (75.5) | 178 | 10 (5.6) |
| 35–44 | 32 | 26 (81.3) | 110 | 6 (5.5) |
| 45–54 | 8 | 6 (75.0) | 28 | 1 (3.6) |
| 55+ | 3 | 2 (66.7) | 16 | 0 |
| Total | 92 | 71 (77.1) | 332 | 17 (5.1) |

**Table 2** Distribution of case and control smears by interval from the index smear[a] and presence of high-risk HPV

| Interval between test and index smear (years) | Case smears | | Control smears | |
|---|---|---|---|---|
| | Number of smears | No. (%) positive for high-risk HPV | Number of smears | No. (%) positive for high-risk HPV |
| <1 | 7 | 6 (85.7) | 6 | 0 |
| 1 | 16 | 12 (75.0) | 104 | 6 (5.8) |
| 2 | 17 | 13 (76.5) | 68 | 2 (2.9) |
| 3 | 24 | 21 (87.5) | 76 | 2 (2.6) |
| 4 | 18 | 12 (66.7) | 45 | 4 (8.9) |
| 5 | 7 | 5 (71.4) | 24 | 1 (4.2) |
| 6 | 3 | 2 (66.7) | 9 | 2 (22.2) |
| Total | 92 | 71 (77.1) | 332 | 17 (5.1) |

[a]‘Index smear’ is the last cytology before diagnosis of CIN for cases and the last available negative smear for controls.

**Table 3** Distribution of case smears by interval before the start of CIN2+ and presence of high-risk HPV

| Interval before start of CIN2+[a] (years) | All smears | | | Excluding smears classified as ASCUS at review[b] | | |
|---|---|---|---|---|---|---|
| | No. of smears | No. (%) HPV positive | | No. of smears | No. (%) HPV positive | |
| < 1 | 22 | 17 (77.3) | 72.26% (95% CI 69.92–86.60%) | 15 | 11 (73.3) | 74.58% (95% CI 62.32–83.97%) |
| 1–2 | 41 | 34 (82.9) | | 28 | 23 (82.1) | |
| 2–3 | 19 | 14 (73.7) | | 13 | 7 (53.9) | |
| 3–4 | 7 | 4 (57.1) | 60.60% (95% CI 26.44–85.86%) | 3 | 2 (66.7) | 71.43% (95% CI 29.14–94.35%) |
| 4–5 | 2 | 1 (50.0) | | 2 | 1 (50.0) | |
| 5–6 | 1 | 1 (100) | | 1 | 1 (100) | |

[a]The midpoint between the last negative cytology and the index smear was used as a proxy for CIN2+ start. [b]Smears classified as ASCUS at review were excluded if no further smear ‘within normal limits’ was available.

 *© 2000 Cancer Research Campaign*

### DISCUSSION

Our results are based on a population putatively representative of cervical screening target groups in industrialized countries. They show that infection from high-risk HPV types is present some years before diagnosis in a high proportion of women who will develop high-grade CIN. According to our data, HPV DNA testing predicts the development of a high-grade lesion 3 years before the conventional diagnosis in some 80% of cases and 6 years before it in 2/3 of cases.

We observed only a slight decrease, if any, in the proportion of HPV-positive smears with an increasing time interval before diagnosis, suggesting that in most cases high-risk HPV infection has a long duration before a high-grade lesion arises.

Decrease remains slight when considering the midpoint between the last negative smear and the index smear as a proxy of the starting date of high-grade lesion. We did not consider that CIN II–III could actually have already been present despite negative cytology confirmed at review. It is not plausible for this to have substantially biased our estimates.

Some 20% of high-grade lesions appear not to be preceded by HPV infection. This figure is similar to the proportion of HPV negative high-grade lesions found in less recent cross-sectional studies (IARC, 1995) although recent studies found a higher sensitivity (Kjellberg et al, 1998; Clavel et al, 1999; Schiffman et al, 2000). Our results, based on archival smears, could underestimate the diagnostic prediction that could be obtained by HPV testing with fresh material.

Controls represent a sample of subjects that remained cytologically negative at least for a time equal to the interval between the test and the index smear. Our data show good specificity of HPV testing since only a small minority of control smears contained high-risk HPV DNA. Such a proportion seems to be fairly stable for the studied test-index smear intervals.

Controls were matched to cases by age, over-representing young ages when compared to the screened population. This could result in an underestimate of overall specificity. Age variation within our controls, although not statistically significant, is in agreement with existing data (Melkert et al, 1993; Burk et al, 1996; Svare et al, 1998). In addition, women with more smears had a higher probability of being selected as controls. A higher frequency of tests could depend on vaginal symptoms, reflecting different sexual behaviour and therefore different probability of HPV infection.

We did not analyse smears for low-risk HPV types. It is possible that some cases that we found negative would test positive for these types. On the other hand this plausibly would also increase the proportion of positive controls, reducing specificity. High specificity is crucial for mass screening.

In our study no obvious selection or information (molecular analysts were blind to case/control status) bias leading to overestimation of the HPV-CIN association is present. Random misclassification of HPV status, if present, is expected to lead to a dilution of such association. We considered multiple smears for some subjects. This could have caused bias in estimates if the number of tests was associated with HPV result (Zeger and Liang, 1986). This is not the case in our study: among the 69 smears from cases with a single test 78.3% were HPV positive against 73.9% of the 23 smears from cases with multiple tests ($P = 0.7$). This, however very small difference was even smaller when adjusting for interval from the index smear.

We studied CINII+ cases arisen in women never referred for colposcopy at previous screening rounds. Women with previous colposcopy history were not suitable for studying the natural history of disease because diagnostic and follow-up procedures could have changed it. In Florence, women with at least low-grade cytology (and until recently with ASCUS) had been referred for colposcopy. This does not mean that study cases did not progress from low-grade lesions: low-grade lesions could have started and progressed to HSIL between screening tests. However, plausibly, the period during which LSIL/ASCUS cytology remained detectable was, on average, shorter in study cases. It is possible that the overall duration of HPV infection before CINII+ development was different in cases previously referred for colposcopy.

A few previous studies considered the risk of cervical invasive or intraepithelial neoplasm subsequent to HPV detection in cytologically normal women. An association between HPV infection and subsequent development of invasive cancer was found by a recent case-control study (Wallin et al, 1999). This study, however, finds a lower proportion of HPV-positive smears before diagnosis (18/48 and 13/35 at time lags <3 years and 3–6 years before cancer diagnosis, respectively) than we do. Studied cancers arose in a screened population: failure to prevent them could have resulted from a more rapid progression than that of prevented cancers. The average duration of pre-invasive lesions before invasive cancer has been estimated to be more than 10 years (Gustafsson and Adami, 1989; Oortmarssen van et al, 1991). In a similar study (Walboomers et al, 1995), among 26 negative archival smears, taken in 18 women developing cervical cancer up to 6 years later, 24 showed HPV infection (23 from high-risk types).

Out of 7 CIN III cases diagnosed in 1622 women in an average follow-up of 40 months after normal cytology, 6 were initially positive to a PCR-based high-risk HPV test (Rozendaal et al, 1996). The 5-year follow-up of a large cohort (Liaw et al, 1999) also found a strong association between HPV infection in cytologically normal women and subsequent SIL. It found a strong increase in risk of SIL (mainly low-grade or ASCUS) in the first few years after HPV infection. Results on high-grade lesions suggested slightly shorter infection duration before high-grade lesions than our data do.

Indirect support to our estimates comes from data on prevalence of HPV infection and of high-grade intraepithelial lesions by age (Schiffman, 1992). The former peak at the beginning of sexual activity (Morrison et al, 1991; Melkert et al, 1993, Burk et al, 1996) and the latter 5–10 ages later, in the early thirties (review in Gustaffson and Adami, 1992).

It has been shown that HPV testing, combined with cytology, can improve sensitivity in detecting the presence of histologically confirmed high-grade lesions (Cuzick et al, 1995, 1999). Recently HPV testing by Hybrid Capture II has been shown to be able to provide better sensitivity (88.4%) than conventional cytology (77.7%) for high-grade lesions, with a specificity (89.0%) acceptable but lower than that of cytology (94.2%) (Schiffman et al, 2000).

Our results suggest that HPV testing could be applied to screening for cervical cancer, by selecting a sub-population with a very low probability of developing a pre-invasive lesion for many years and another with a relatively high probability. The former could safely be invited for a new test at long intervals while the latter could be followed up at short intervals. The detection rate of histologically confirmed high-grade CIN in the Florence screening is 2.2 per thousand. Applying our data to such a situation, 350 per

© 2000 Cancer Research Campaign 

10 000 women with high-risk HPV DNA will have a CIN II+ diagnosis at the next 3-year screening round compared to 4.5 per 10 000 in HPV-negative women. The mentioned detection rate includes cases arisen in women with a colposcopy history at previous rounds while our 'sensitivity' estimates do not (see above). Therefore a slight over- or underestimate is possible.

In a simulation study (Ballegoijen van et al, 1997), when a 10-year duration of infection before progression to CIN was assumed, 10-yearly HPV testing provided greater protection than 3-yearly conventional cytology. Outcomes were the opposite when assuming a 1-year duration. At that time the authors could not reach a conclusion because available studies did not allow reliable assumptions for this parameter. Our results suggest long duration and, therefore, good cost-effectiveness in screening for cervical cancer based on long-interval HPV testing. Caution is however needed.

### ACKNOWLEDGEMENTS

This study was partially financially supported by EC 'Europe Against Cancer' contract SOC97 20114205FO2. We thank J Cuzick for thoughtful comments.

### REFERENCES

Ballegoijen M Van, Akker van Marle ME Van den, Warmerdam PG, Meijer CJLM, Walboomers JMM and Habbema JDF (1997) Present evidence on the value of HPV testing for cervical cancer screening: a model-based exploration of the (cost-) effectiveness. *B J Cancer* **76**: 651–657

Brookmeyer R, Liang KY and Linet M (1986) Matched case-control designs and overmatched analyses. *Am J Epidemiol* **124**: 693–701

Burk RD, Kelly P, Feldman J, Bromberg J, Vermund SH, DeHovitz JA and Landesman SH (1996) Declining prevalence of cervicovaginal human papillomavirus infection with age is independent of other risk factors. *Sex Transm Dis* **23**: 333–341

Clavel C, Masure M, Bory JP, Mangeonjean C, Lorenzato M, Gabriel R, Quereux C and Birembaut P (1999) Hybrid capture II-based human papillomavirus detection, a sensitive test to detect in routine high grade cervical lesions: a preliminary study on 1518 women. *B J Cancer* **80**: 1306–1311

Confortini M, Biggeri A, Cariaggi MP, Carozzi FM, Minuti PA, Russo A and Palli D (1993) Intralaboratory reproducibility in cervical cytology. Results of the application of a 100-slide set. *Acta Cytol* **37**: 49–54

Cuzick J (2000) Human papillomavirus testing for primary cervical cancer screening. *JAMA* **283**: 108–109

Cuzick J, Szarewski A, Terry G, Ho L, Hanby A, Maddox P, Anderson M, Kocjan G, Steele ST and Guilleband J (1995) Human papillomavirus testing in primary cervical screening. *Lancet* **345**: 1533–1536

Cuzick J, Beverley E, Ho L, Terry G, Sapper H, Mielzynska, Lorincz A, Chan WK, Krausz T and Soutter P (1999) HPV testing in primary screening of older women. *BJ Cancer* **81**: 554–558

Fujinaga Y, Shimada M, Okazawa K, Fukushima M, Kato I and Fujinaga K (1991) Simultaneous detection and typing of genital human papilloma virus DNA using the polymerase chain reaction. *J General Virol* **72**: 1039–1044

Gustafsson L and Adami HO (1989) Natural history of cervical neoplasia: consistent results obtained by an identification technique. *Br J Cancer* **60**: 132–141

Gustaffson L and Adami HO (1992) Optimisation of cervical cancer screening. *Cancer Causes and Control* **3**: 125–136

Ho GYF, Burk RD, Klein S, Kadish AS, Chang CJ, Palan P, Basu J, Tachezy R, Lewis R and Romney S (1995) Persistent genital human papillomavirus infection as a risk factor for persistent cervical dysplasia. *J Natl Cancer Inst* **87**: 1365–1371

International Agency for Research on Cancer (1995) Human Papillomaviruses. IARC Monographs on the evaluation of carcinogenic risks to humans **64**: 133–134, Lyon

Kjellberg L, Wilklund F, Sjöberg I, Wadell G, Ångström T, Dillner J and Mählck CG (1998) A population-based study of human papillomavirus deoxyribonucleic acid testing for predicting cervical intraepithelial neoplasia. *Am J Obstet Gynecol* **179**: 1497–1502

Liaw KL, Glass AG, Manos MM, Greer CE, Scott DR, Sherman M, Burk RD, Kurman RJ, Wacholder S, Rush BB, Cadell DM, Lawler P, Tabor D and Schiffman M (1999) Detection of human papillomavirus in cytologically normal women and subsequent cervical squamous intraepithelial lesions. *J Natl Cancer Inst* **91**: 954–960

Meijer CJLM, Brule AJC van den, Snijders PJF, Helmerhorst T, Kenemans P and Walboomers JMM (1992) Detection of human papillomavirus in cervical scrapes by the polymerase chain reaction in relation to cytology: possible implications for cervical cancer screening. In: Munoz N, Bosch FX, Shah KV, Meheus A (eds) *The epidemiology of cervical cancer and human papillomavirus*. IARC Scientific Publications No 119: Lyon, IARC

Melkert PWJ, Hopman E, van den Brule AJC, Risse EKI, van Diest PJ, Bleker OP, Hlemerhorst T, Schipper EI, Meijer CJLM and Walboomers JMM (1993) Prevalence of HPV in cytomorphologically normal cervical smears, as determined by polymerase chain reaction, is age-dependent. *Int J Cancer* **53**: 919–923

Morrison EAB, Ho GYF, Vermund SH, Goldberg GL, Kadish AS, Kelley KF and Burk RD (1991) Human papillomavirus infection and other risk factors for cervical neoplasia: a case-control study. *Int J Cancer* **49**: 6–13

Nobbenhuis M, Walboomers JMM, Helmerhorst TJM, Rozendaal L, Remmink AJ, Risse EKJ, van der Linden HC, Voorhorst FJ, Kenemans P and Meijer CJHLM (1999) Relation of human papillomavirus to cervical lesions and consequences for cervical cancer screening: a prospective study. *Lancet* **354**: 20–25

Oortmarssen GJ Van and Habbema JDF (1991) Epidemiological evidence for age-dependent regression of pre-invasive cervical cancer. *BJ Cancer* **64**: 559–565

Palli D, Carli S, Cecchini S, Venturini A, Piazzesi G and Buiatti E (1990) A centralised cytologic screening programme for cervical cancer in Florence. *J Epidemiol Community Health* **44**: 47–51

Palli D, Confortini M, Biggeri A, Russo A, Cariaggi MP and Carozzi MF (1993) A quality control system involving peer review of abnormal cervical smears. Cytopathology **4**: 17–25

Reid R, Greenberg MD, Lorincz A, Jenson AB, Laverty CR, Husain M et al (1991) Should cervical cytology testing be augmented by cervicography or human papillomavirus deoxyribonucleic acid detection? *Am J Obstet Gynecol* **164**: 1461–1471

Remmink AJ, Walboomers JMM, Helmerhorst TJM, Voorhorst FJ, Rozendaal L, Risse EKJ, Meijer CJLM and Kenemans P (1995) The presence of persistent high-risk HPV genotypes in dysplastic cervical lesions is associated with progressive disease: natural history up to 36 months. *Int J Cancer* **61**: 306–311

Richart RM (1995) Screening. The next century. *Cancer* **76**: (10 suppl): 1919–1927

Roda Husman AM de, Snijders PJ, Stel HV, Brule AJC van den, Meijer CJLM and Walboomers JMM (1995) Processing long-stored archival cervical smears for human papillomavirus detection by the polymerase chain reaction. *Br J Cancer* **72**: 412–417

Ronco G and Biggeri A (1999) Estimating sensitivity and specificity when repeated tests are performed on the same subject. *J Epidemiol Biostatistics* **4**: 329–336

Rozendaal L, Walboomers JMM, Linden JC van der, Voorhorst FJ, Kenemans P, Helmerhorst ThJM, Ballegooijen M Van and Meijer CJLM (1996) PCR-based high-risk HPV test in cervical cancer screening gives objective risk assessment of women with cytomorphologically normal cervical smears. *Int J Cancer* **68**: 766–769

Schiffman MH (1992) Recent progress in defining the epidemiology of Human Papillomavirus infection and cervical neoplasia. *J Natl Cancer Inst* **84**: 394–398

Schiffman MH, Herrero R, Hildesheim A, Sherman ME, Bratti M, Wacholder S, Alfaro M, Hutchinson M, Morales J, Greenberg MD and Lorincz AT (2000) HPV DNA testing in cervical cancer screening: results from women in a high-risk province of Costa Rica. *JAMA* **283**: 87–93

Solomon D (Editorial) (1993) Screening for cervical cancer: prospects for the future. *J Natl Cancer Inst* **85**: 1019

Svare EI, Kjaer SK, Smits HL, Poll P, Tjong-A-Hung SP and ter Schegget J (1998) Risk factors for HPV detection in archival smears. A population based study from Greenland and Denmark. *Europ J Cancer* **34**: 1230–1234

Walboomers JMM, Roda Husman AM de, Snijders PJF, Stel HV, Risse EK, Helmerhorst TJ, Voorhorst FJ and Meijer CJLM (1995) Human papillomavirus

 *© 2000 Cancer Research Campaign*

in false negative archival cervical smears: implications for screening for cervical cancer. *J Clin Pathol* **48**: 728–732

Wallin KL, Wilklund F, Ångström T, Bergman F, Stendhal U, Wadell G, Hallmans G and Dillner J (1999) Type specific persistence of human papillomavirus DNA before the development of invasive cervical cancer. *N Engl J Med* **341**: 1633–1638

Wright TC Jr, Denny L, Kuhn L, Pollack A and Lorincz A (2000) HPV DNA testing of self-collected vaginal samples compare with cytologic screening to detect cervical cancer. *JAMA* **283**: 81–86

Zeger SL and Liang KY (1986) Longitudinal analysis for discrete and continuous outcomes. *Biometrics* **42**: 121–130

*© 2000 Cancer Research Campaign*
